# Discrimination and isolation of the virus from free RNA fragments for the highly sensitive measurement of SARS-CoV-2 abundance on surfaces using a graphene oxide nano surface

**DOI:** 10.1186/s40580-021-00281-8

**Published:** 2021-10-18

**Authors:** Hyun Jin Yoo, Yun Guang Li, Wen Ying Cui, Wonseok Chung, Yong-Beom Shin, Yeon-Sook Kim, Changyoon Baek, Junhong Min

**Affiliations:** 1 School of Integrative Engineering, Chung-Ang University, Heukseok-dong, Dongjak-gu, Seoul, 06974 South Korea; 2 BioNano Health Guard Research Center, Daejeon, 34141 South Korea; 3 Division of Infectious Diseases, Department of Internal Medicine, Chungnam National University School of Medicine, Munhwa-ro 282, Jung-gu, Daejeon, 35015 South Korea

**Keywords:** SARS-CoV-2, Graphene oxide, Virus isolation, Molecular diagnostics

## Abstract

It is highly important to sensitively measure the abundance of severe acute respiratory syndrome coronavirus 2 (SARS-CoV-2) on various surfaces. Here, we present a nucleic acid-based detection method consisting of a new sample preparation protocol that isolates only viruses, not the free RNA fragments already present on the surfaces of indoor human-inhabited environments, using a graphene oxide-coated microbead filter. Wet wipes (100 cm^2^), not cotton swabs, were used to collect viruses from environmental surfaces with large areas, and viruses were concentrated and separated with a graphene oxide-coated microbead filter. Viral RNA from virus was recovered 88.10 ± 8.03% from the surface and free RNA fragment was removed by 99.75 ± 0.19% from the final eluted solution. When we tested the developed method under laboratory conditions, a 10-fold higher viral detection sensitivity (Detection limit: 1 pfu/100 cm^2^) than the current commercial protocol was observed. Using our new sample preparation protocol, we also confirmed that the virus was effectively removed from surfaces after chemical disinfection; we were unable to measure the disinfection efficiency using the current commercial protocol because it cannot distinguish between viral RNA and free RNA fragments. Finally, we investigated the presence of SARS-CoV-2 and bacteria in 12 individual negative pressure wards in which patients with SARS-CoV-2 infection had been hospitalized. Bacteria (based on 16 S DNA) were found in all samples collected from patient rooms; however, SARS-CoV-2 was mainly detected in rooms shared by two patients.

**Supplementary Information:**

The online version contains supplementary material available at 10.1186/s40580-021-00281-8.

## Introduction

The outbreak of coronavirus disease of 2019 (COVID-19), caused by severe acute respiratory syndrome coronavirus 2 (SARS-CoV-2), began in China at the end of 2019 and has become a pandemic [1, 2]. As of the end of June 2021, the number of confirmed cases worldwide had exceeded 180.4 million, and more than 3,900,000 people had died [3]. Antiviral drugs and vaccines for SARS-CoV-2 are presently being developed worldwide, but perfect therapeutics and vaccines are still challenging due to the continued mutation of virus [4–7]. Currently, the best method to prevent virus transmission is to rapidly diagnose and quarantine asymptomatic and symptomatic patients and completely disinfect the virus-contaminated environment [8–11].

Nucleic acid (NA)-based diagnosis, using ribonucleic acid (RNA) amplification, is currently one of the most popular methods to measure the viral abundance in both patients with suspected infections and the environment, because this method is more sensitive than antigen or antibody-based tools as it directly utilizes and amplifies the target viral RNA [12, 13]. Nevertheless, the gravity of the pandemic has increased because of the lack of rapid and accurate means of virus detection for point-of-care tests [14, 15], which is a reflection of the limitations of current sample preparation technologies. To increase the accuracy (sensitivity and specificity) of pathogen detection, it is important to develop appropriate sample preparation techniques to not only collect the target virus (or bacteria) but also extract and purify NA [deoxyribonucleic acid (DNA) or RNA].

Swab protocols utilizing guanidine-based technology are presently the most commonly used method of collecting target pathogens from patients or environments and extracting and purifying NA [16–18]. However, there are serious drawbacks to this swab protocol, which include the following: (1) Cotton swabs are suitable for collecting viruses from the throat or nasal cavity, but they are too small to collect viruses in living environments over large surfaces. (2) In addition to intact viruses, free RNA fragments released from already lysed viruses are also collected from patients or the environment, which can lead to false positive signals. This is because RNA exposed to the environment is stable for more than 24 h (Additional file 1: Figure S1).

Graphene-based nanomaterials have been widely used in various technologies due to their unique properties and nanoscale dimensions [19–21]. In particular, graphene oxide (GO) is of interest for applications in the fields of medicine, biotechnology, and various interdisciplinary fields, owing to the properties associated with its several functional groups, which include oxygen, epoxide, carbonyl, and hydroxyl groups [22]. GO has been utilized to adsorb biomaterials, such as NAs, proteins, and bacteria, because it has both aromatic (sp^2^) and aliphatic (sp^3^) domains that facilitate interactions at its surface interface [23, 24]. To increase the sensitivity of pathogen detection, we developed bacterial and viral concentration procedures using GO-grafted microbeads in our previous study [25]. However, this technique did not discriminate between NA from pathogens and free NA that already existed on surfaces.

In the present study, we first developed an effective sample preparation technology that can be used to directly measure the abundance of highly infectious pathogens on suspected contaminated surfaces by isolating only pathogens, not free NA, using nanostructured GO surfaces. The interactions of viruses and free RNA fragments on the GO surface were compared, with respect to their pH-dependent adsorption and desorption processes. This sample preparation tool was then applied to environmental monitoring inside negative pressure wards where COVID-19 patients were undergoing treatment.

**Fig. 1:**
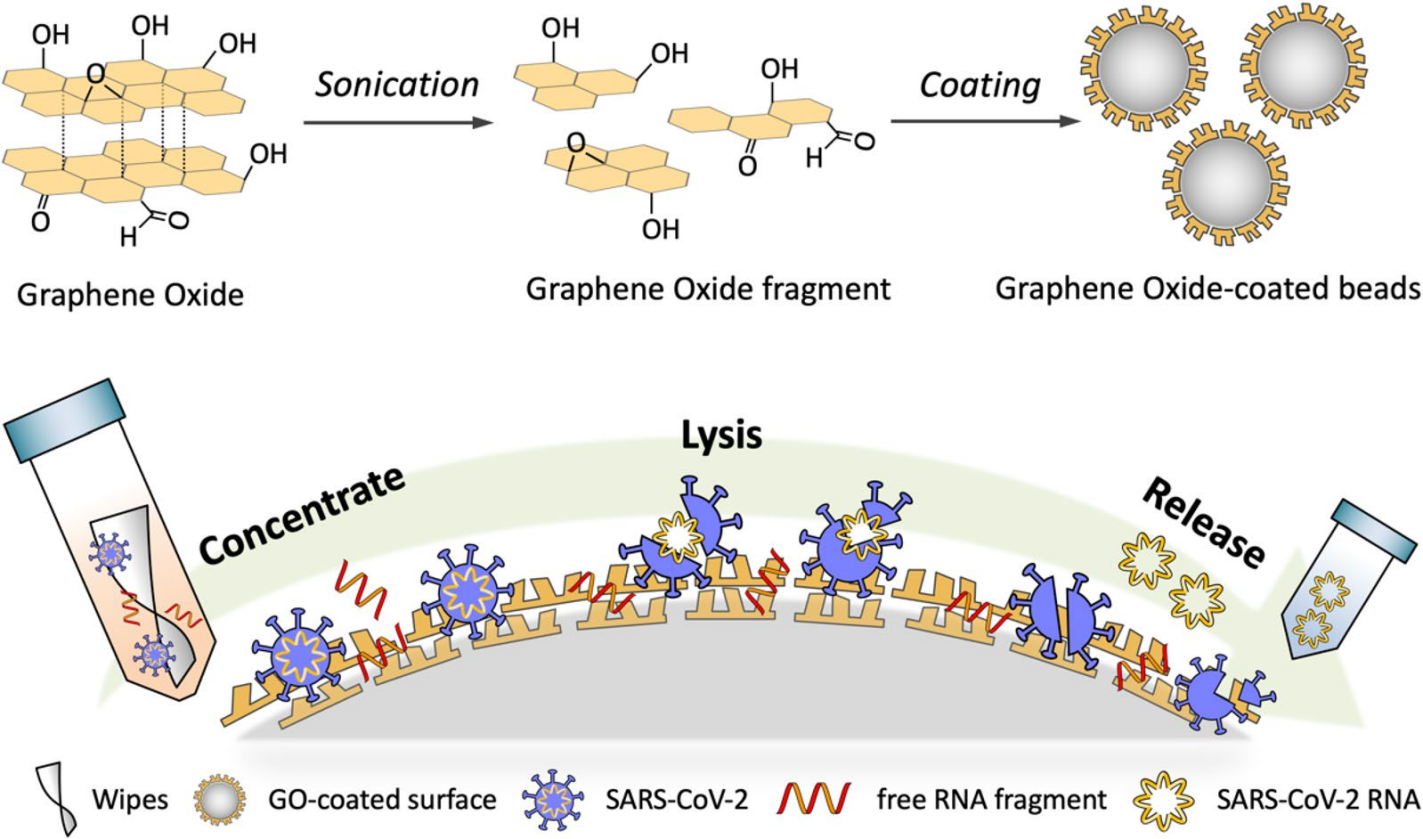
Schematic illustration of GO based sample preparation system for nucleic acid-based detection of SARS-CoV-2, not free RNA fragments

In Fig. 1, pathogens (bacteria and SARS-CoV-2) and free RNA fragment were collected from a large, potentially contaminated surface (e.g., patient bed, ward wall, lavatory, and cell phone) using wipes. Both the infectious pathogens and free RNA fragments were adsorbed onto nanostructured GO surface at low-pH condition. The adsorbed pathogens were lysed using physical and chemical methods, and their NA were released at high-pH condition. The extracted NA at high-pH condition were not adsorbed to the nanostructured GO surface, whereas free RNA fragments, which had been adsorbed on the nanostructured GO surface at low-pH condition, remained attached at high-pH condition. Therefore, only RNA released from pathogens at the lysis step, not free RNA fragments, were eluted into the final solution. The sample was loaded onto a real time polymerase chain reaction (PCR) system to identify SARS-CoV-2 or total bacteria using appropriate primer sets without the potential for false positive signals induced by free RNA fragments.

## Methods/experimental

### Materials

Poly(ethylene glycol) (PEG) 8000 and Trizma^®^ base were purchased from Sigma-Aldrich Co. (MO, US). General use chemicals were obtained from Junsei-Chemical (Tokyo, Japan). Glass microbeads (~40–70 μm in diameter) were obtained from Daihan Scientific (South Korea). The artificial surface in the laboratory was prepared using a 1 m × 1 m-sized acrylic plate (poly (methyl methacrylate); PMMA). MD-125 (MICROG ENKOREA, South Korea), mainly composed of alkyldimethylbenzylammonium chloride, was used as a disinfectant.

### Nanostructured GO-coated microbeads

GO was synthesized using Hummer’s method with some modifications. Briefly, 1 g of graphite powder (Sigma-Aldrich) was added to 23 mL of H_2_SO_4_ and vigorously stirred for 12 h. Then, 3 g of KMnO_4_ (Sigma-Aldrich) was added to the suspension and incubated at 35 °C for 30 min. The temperature was increased up to 70 °C and maintained for 45 min. Next, 140 mL of distilled water and 10 mL of 30% H_2_O_2_ were added, and the mixture was stirred for 1 h. The synthesized GO was rinsed with 5% HCl and distilled water. The glass beads, which were cleaned with piranha solution, were immersed in (3-aminopropyl) triethoxysilane (98%; Sigma-Aldrich) containing 95% ethanol solution for 1 h at room temperature and then heated at 120 °C for 2 h. The pretreated beads were immersed in GO-dispersed ethanol solution for 2 h. GO-dispersed ethanol was prepared via ultrasonication (150 W) treatment.

The Fourier-transform infrared (FTIR) spectroscopy results were obtained using FTIR-ALPHA II (Bruker, MA, USA) within the wavenumbers ranging from 500 to 4000 cm^−1^ with 64 scans. The X-ray photoelectron spectroscopy (XPS) spectrum of the carbon region was measured by using K-alpha plus XPS (Thermo Fisher Scientific, MA, USA). The Nanoscope IV/Multimode Atomic force microscope (AFM) device (Digital Instruments, USA) was used to investigate the nanostructures and GO topography of the coated surface.

### Human coronavirus 229E (HCoV-229E) cultivation

HCoV-229E was provided by the BioNano Health-Guard Research Center. MRC-5 (ATCC, Manassas, VA, USA) cells were grown to a concentration of 1 × 10^6^ cells in a 25 cm^2^ cell culture flask (SPL, South Korea) containing growth medium supplemented with 10% fetal bovine serum (FBS, Gibco, Grand Island, NY, USA) and 1% antibiotics (Gibco). Prior to the inoculation of the cells with the virus, the growth medium was removed. The virus [virus culture medium was Dulbecco’s MEM containing 2% FBS, 1:10(v/v)] was inoculated and incubated with shaking every 10 min for 1 h at 37 °C in 5% CO_2_. The virus culture medium was then removed. The infected MRC-5 cells were cultivated in 4 mL of Dulbecco’s MEM containing 2% FBS at 37 °C in 5% CO_2_ for 5 days. When 90% of the cells detached from the bottom, the virus was isolated by centrifugation at 3200×*g* for 10 min.

### New sample preparation protocol and current commercial protocol

Wet wipes (10 cm × 10 cm) were used to collect the virus from the surface (1 m × 1 m). After collection, the wipes were soaked in 10 mL of desorption buffer (Tris-HCl buffer (10 mM, pH 9) with 5% ethanol) and shaken for 1 min, such that the collected virus was transferred to the desorption buffer. When 1 mL of adsorption buffer (1 M sodium acetate buffer, pH 5) was added to the recovered desorption buffer, the virus was adsorbed onto the GO surface as the solution was passed through the GO bead column by centrifugation (250×*g*, 3 min). The RNA from the virus adsorbed onto the GO surface was extracted by adding 300 µL of lysis buffer (50 mM NaOH and 5% PEG 8,000) into the column and soaking for 3 min. The solution containing the extracted RNA was released into the elution tube by centrifugation (250×*g*, 3 min).

The current commercial method, which involves using swabs to collect viruses present on surfaces, was also used to collect the virus from the experimental surface (1 m × 1 m). After collection, the swab was soaked in 200 µL of phosphate buffer saline and shaken for 1 min. It was treated with a QIAamp viral RNA mini kit (Qiagen, Hilden, Germany) for RNA extraction.

### RT-qPCR and PCR assay

RT-qPCR was performed to analyze the RNA of SARS-CoV-2, HCoV-229E, and Influenza A virus subtype H1N1 on a thermocycler (LightCycler^®^ 480 Instrument II, Roche, Basel, Switzerland); reactions were assembled with TaqPath™ 1-Step RT-qPCR Master Mix (Applied Biosystems, CA, USA).

The total amounts of bacteria were determined by qPCR DNA quantification of the 16 S DNA gene using identical equipment and TB Green ® Premix Ex Taq (Takara, Japan). Detailed information regarding nucleic acid amplification is summarized in Additional file 1: Table S1 [26–29].

### Artificial virus-contaminated surfaces

A bare surface (PMMA, 1 m^2^) was cleaned with 70% ethanol solution three times and sterilized with UV light (50 W) for 30 min. A target solution, containing various concentrations of a combination of intact HCoV-229E virus (to represent viral RNA) or Influenza A virus subtype H1N1 RNA (to represent free RNA fragments, Fig. 3), was evenly spread on the sterilized bare surface and dried for 30 min at room temperature and under 30% relative humidity.

### Field test

A field test was performed in the negative pressure wards at Chungnam National University (Daejeon, South Korea) in which COVID-19 patients were hospitalized. Four sampling points (bed frame, toilet in lavatory, wall, and personal cell phones of the patients) in the negative pressure ward were selected [30, 31]. Samples were collected 24 h after the disinfection process. SARS-CoV-2 RNA from each sample was obtained using the new sample preparation kit and amplified using RT-qPCR.

## Results and discussion

### Surface modification with graphene oxide

**Fig. 2:**
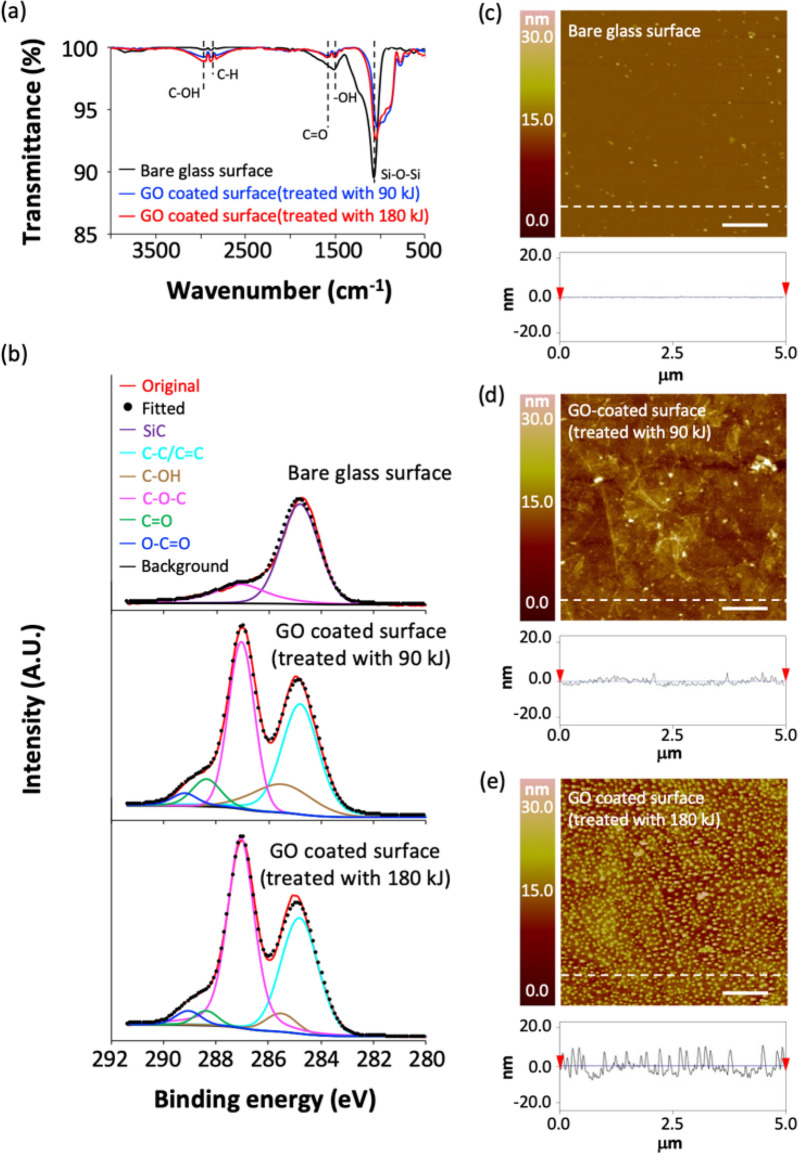
Analysis of the GO-coated surface by **a** FTIR spectra, and **b** XPS spectrum of the carbon region. AFM image of **c** bare glass surface, **d** GO-coated surface treated with 90 kJ, and **e** GO-coated surface treated with 180 kJ. (Scale bar = 1 µm)

Glass beads were coated with GO to separate viral RNA and free RNA fragment. Surface modification was confirmed with FTIR, XPS and AFM. The FTIR spectra of bare glass surface and GO-coated surface were shown in Fig. 2a. The adsorption peak at around 1070 cm^−1^ corresponding to Si–O–Si were detected among all of samples and was decreased by GO coating. Peaks corresponding to the carbon groups (C=O at 1710 cm^−1^, C–H at 2870 cm^−1^ and C–OH at 3450 cm^−1^) were observed at both of GO-coated surfaces, however there were no significant difference depending on the GO treatment conditions [32, 33].

The XPS spectrum of the carbon region (C1s) was used to confirm the chemical composition of the surface, as shown in Fig. 2b. Bare glass surface had SiC peak at 848.8 eV and also C–O–C peak was observed. GO-coated surface has 5 peaks which corresponding to the following functional groups (C–C/C=C at 284.8 eV, C–OH at 285.5 eV, C–O–C at 287.0 eV, C=O at 288.4 eV and O–C=O at 289.2 eV) [34, 35]. It can be seen that the major peak of GO-coated surface is epoxy/ether group (C–O–C). As expected, composition of functional groups in both GO surfaces were no significant difference.

The morphology of GO-coated surface was confirmed with AFM image, as shown in Fig. 2c–e. The GO-coated surface treated with 90 kJ (Fig. 2d) showed that GO was coated on the surface in the form of a plate. On the other hand, it can be observed that the surface coated with GO treated with 180 kJ can be seen that the nanostructured GO is on the surface (Fig. 2e). Analyzing the roughness of the surface, the surface coated with GO treated with 90 kJ had Ra: 1.711 nm and Rq: 2.432 nm, whereas the surface coated with GO treated with 180 kJ had Ra: 3.147 nm and Rq: 3.956 nm. It implies morphological structure was changed whereas chemical properties of GO were not changed by sonication process.

### Isolation of coronavirus RNA from free RNA fragments

**Fig. 3:**
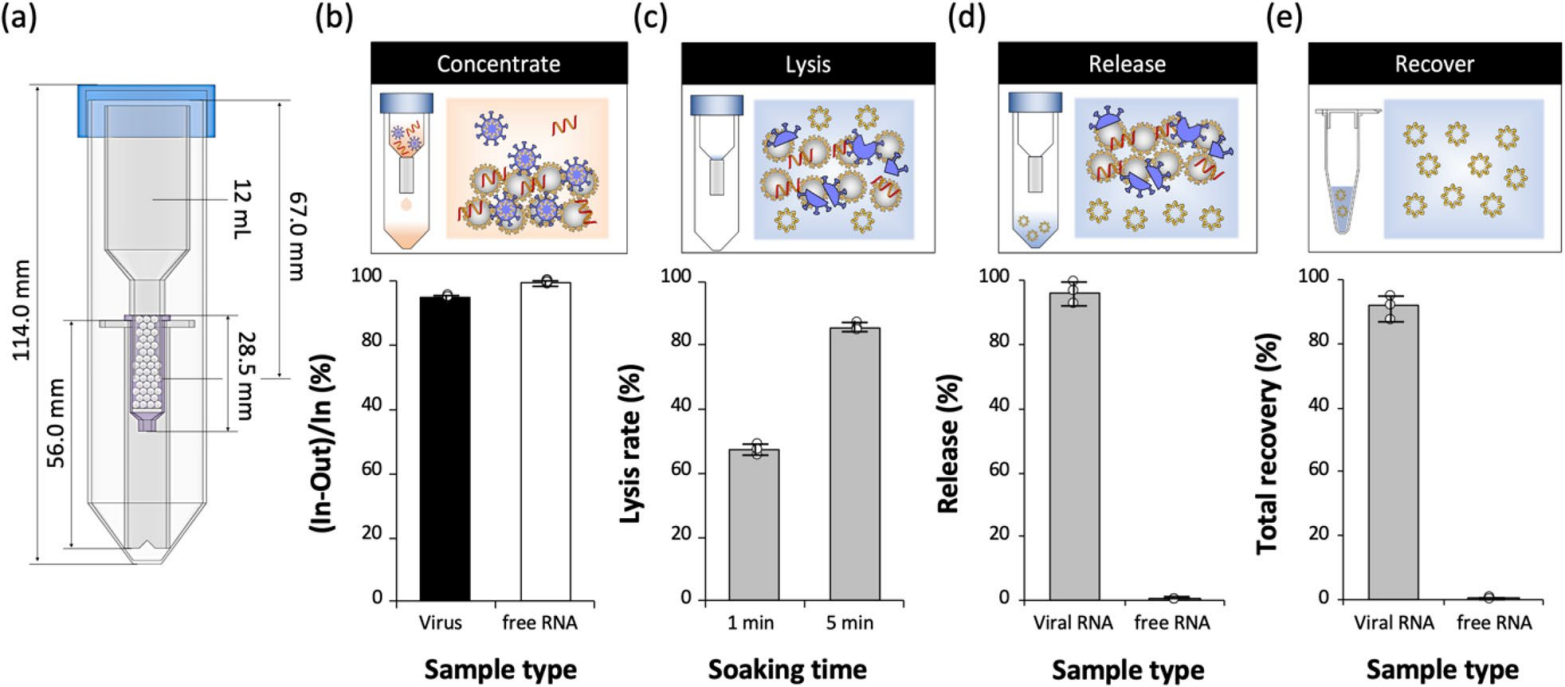
New sample preparation protocol and kit to isolate only target RNA in virus, not free RNA fragments; **a** sample tube and filter column consisting of GO-coated microbeads, **b** adsorption efficiency (%) of virus and free RNA fragments on the GO surface, **c** virus lysis efficiency, **d** input-based RNA amount (%) in the solution (i.e., the amount of RNA released from virus and free RNA fragments), and **e** total recovery (%) of viral RNA and free RNA fragments through the whole process

GO-coated glass beads (~ 40–70 µm, 0.6 g) were packed into the isolation column as shown in Fig. 3a. The column was designed to directly apply to commercial sample tubes and centrifuges. An artificial sample containing coronavirus (HCoV-229E) and free RNA fragments (from Influenza A virus subtype H1N1) was introduced and translocated through the isolation column by centrifugation (250×*g*). Most of the virions and free RNA fragments were adsorbed onto the GO surface as shown in Fig. 3b. This finding was consistent with other previous results in which nanostructured GO, with various functional groups on the aliphatic and aromatic domains, has better binding properties than silica surfaces [25]. RNA was successfully released from the virus using a simple chemical lysis solution (Fig. 3c), and a 5 min soaking time in this solution at room temperature was determined to be the optimal conditions for RNA release. After direct elution of the lysis buffer solution by centrifugation, the amount of RNA recovered in the buffer solution was measured; the results are presented in Fig. 3d. Interestingly, the RNA released from the virus upon viral lysis was not adsorbed onto GO at high pH (lysis solution), whereas the free RNA fragments, which had adsorbed onto GO at low pH, was not desorbed from GO at high pH. It is likely that the binding energy between RNA and GO would be higher than that between RNA and silica, which is normally used in commercial sample preparation tools [36]. Consequently, the total recovery efficiencies for virus RNA and free RNA fragments in the whole sample preparation steps were 88.10 ± 8.03% and 0.25 ± 0.19%, respectively, as shown in Fig. 3e.

### Investigation of surfaces artificially contaminated with HCoV-229E

**Fig. 4:**
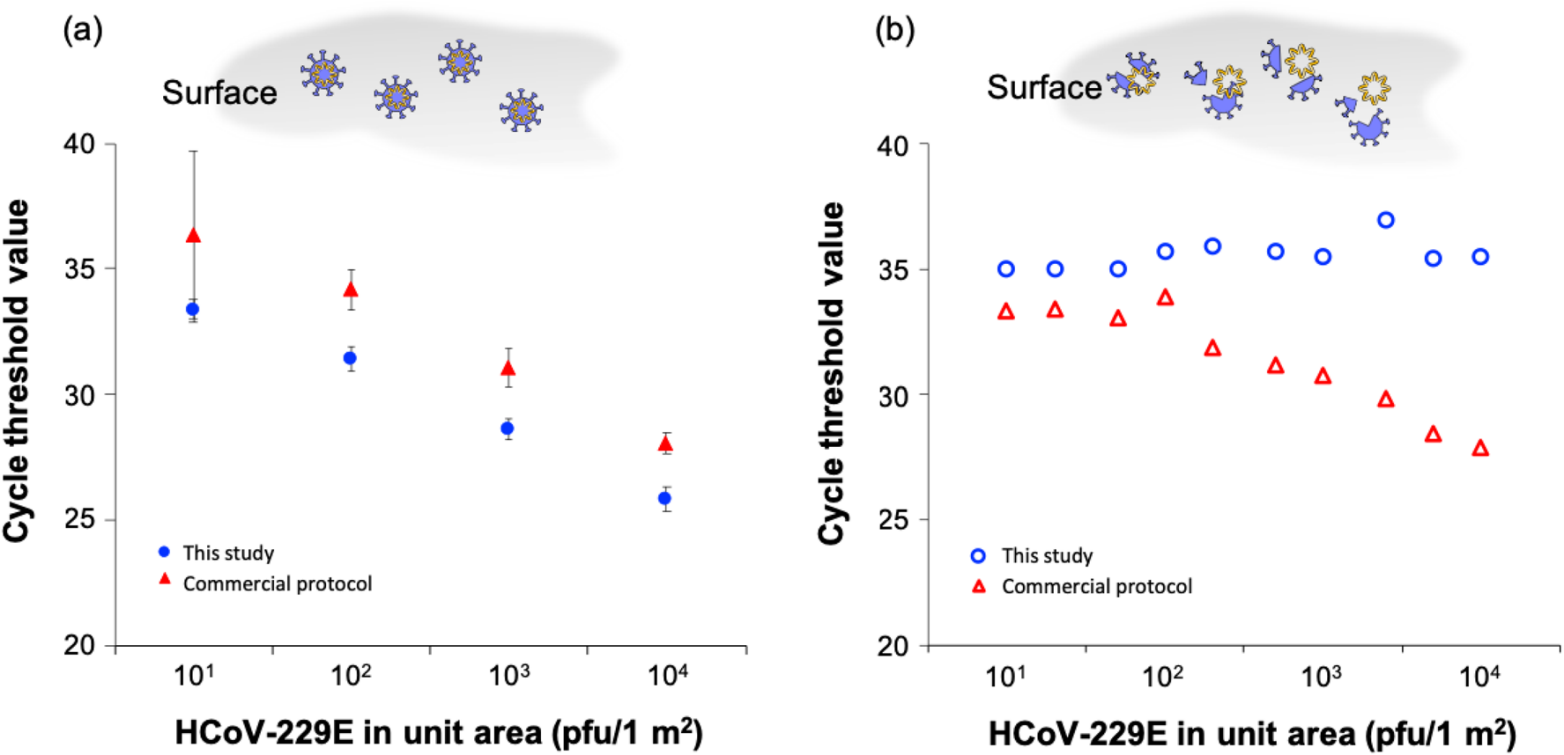
RT-qPCR results for various HCoV-229E solution amounts using the new sample preparation protocol and current commercial protocol **a** on the normal test surface and **b** a test surface disinfected by chemicals

To confirm the detection efficiency of the new protocol developed in the current study, various amounts of HCoV-229E solution (10^1^–10^4^ pfu) were spread on a PMMA surface (1 m^2^) and fully dried for 30 min. As shown in Fig. 4a, the RT-qPCR cycle threshold values for samples prepared with the new sample preparation protocol were lower than those prepared with the guanidine-based current commercial protocol (QIAamp viral RNA mini kit with swab) [37–39]. This result implies that the detection sensitivity of our newly developed protocol was approximately 10-fold higher than that of current commercial tools. Furthermore, wet wipes may also be more effective in collecting the virus over a large area than a normal swab. The performance of our new protocol was also confirmed with SARS-CoV-2 as shown in Additional file 1: Figure S2. The cycle threshold value with our new protocol decreased by 1.97 relative to the cycle threshold using the current commercial protocol.

Figure 4b shows the results for PCR-based molecular measurements of viral abundance on artificially viral-contaminated surfaces that were treated by a disinfectant mainly composed of alkyldimethylbenzylammonium chloride [40, 41]. The qPCR cycle threshold value for surfaces that had been contaminated with HCoV-229E and then decontaminated was higher than 35 (i.e., a negative result) when our new sample preparation protocol was employed, whereas the qPCR results for samples prepared with the current commercial sample preparation protocols were similar to those for the non-disinfected virus-contaminated surfaces, as shown in Fig. 4a. This result implies that (1) the disinfectant composed of alkyldimethylbenzylammonium chloride is very effective at sanitizing HCoV-229E-contaminated surfaces, (2) RNA is not degraded by the disinfectant, and (3) the new sample preparation protocol developed in this study can isolate RNA released from the virus and adsorbed on nanostructured GO surface from the free RNA fragments that are present on environmental surfaces.

### Filed tests

This new sample preparation protocol was introduced to measure SARS-CoV-2 surface contamination in an environmental test at the negative pressure ward in a hospital where COVID-19 patients were hospitalized. The performance of the new sample preparation protocol was compared to that of the current commercial protocol using environmental samples collected before and after disinfection. The samples were collected from the patient beds, walls, and toilets (seats) in negative pressure wards (Additional file 1: Figure S3). For each sample, RNA was prepared using two protocols (new protocol and current commercial protocol), which were analyzed by RT-qPCR, as shown in Table 1. In the case of the new sample preparation protocol, the RT-qPCR cycle threshold values for the wall samples increased by 2.3 after environmental disinfection. In contrast, there was no difference in cycle threshold values before and after disinfection when the guanidine-based commercial protocol was utilized.

**Table 1:** Differences in RT-qPCR results (cycle threshold value) using two different sample preparation methods (the new protocol developed in the current study and standard commercial protocol) for test surfaces on which SARS-CoV-2 had been spread

Sites	Disinfection methods	This study			Commercial protocol		
		Cycle threshold value (before disinfection)	Cycle threshold value (after disinfection)	∆Cycle threshold value	Cycle threshold value (before disinfection)	Cycle thresholdvalue (after disinfection)	∆Cycle threshold value
Bed	Spray	40.00	40.00	0.00	36.61	36.57	− 0.04
Room wal	Spray	37.70	40.00	2.30	36.26	36.32	0.06
Inner lavatory	Spray-mopping	33.50	40.00	6.50	34.91	38.55	3.64

Samples were collected from test surfaces before and after disinfection

This implies that only virus RNA, not free RNA fragments, was extracted using the new protocol, whereas the guanidine-based commercial protocol does not discriminate between the viruses and free RNA fragments. These results were confirmed in the lavatory (toilet seat) samples collected in negative pressure wards. When free RNA fragments were largely removed by mopping, the cycle threshold value for the inner lavatory samples increased significantly, regardless of the sample preparation method. This also implies that on the patient bed, although viruses were not found, free RNA fragments pre-released from SARS-CoV-2 were present.

**Fig. 5:**
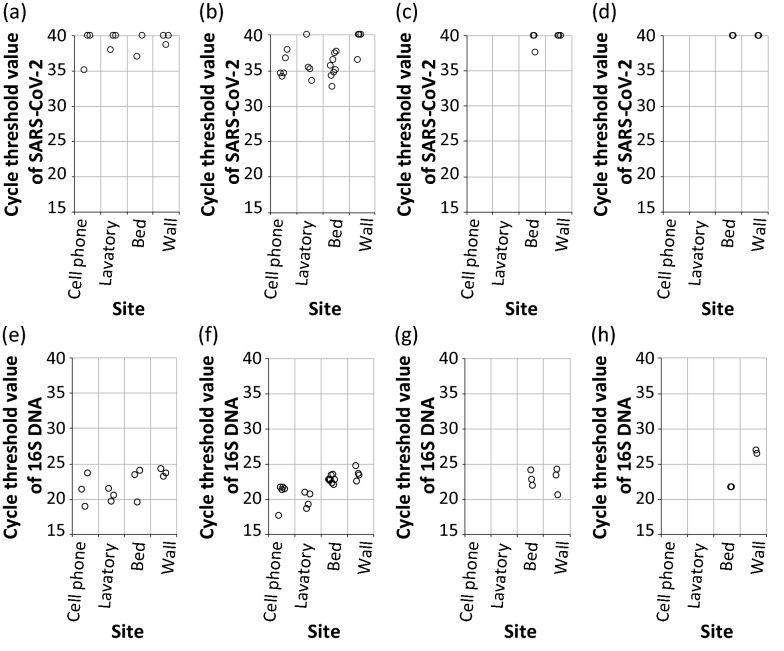
RT-qPCR results of SARS-CoV-2 and qPCR results of total bacteria in environmental samples collected from surfaces in various negative pressure COVID-19 patient wards; **a**, **e** single-person negative pressure wards in which patients can move; **b**, **f** double-shared (two-person) negative pressure wards (patients can move); **c**, **g** single-person negative pressure wards within an intensive care unit (patients with nasal cannula unit cannot move); **d**, **h** single-person negative pressure wards within an intensive care unit (patients with extubation unit cannot move)

Using the new sample preparation protocol consisting of wet wipes and a GO bead column, we investigated 12 individual negative pressure wards (not disinfected for 24 h) in which patients had been hospitalized, to detect SARS-CoV-2 and total bacteria. As shown in Fig. 5, three single-person negative pressure wards (a, e), four double-sharing (two-person) negative pressure wards (b, f), and five single-person negative pressure wards for intensive care (c and d, g and h) were selected (also see Additional file 1: Figure S3). Samples were collected from patient cell phones, toilets, bed frames, and walls. We did not investigate cell phones and toilets in intensive care rooms because patients could not use them at all. Viruses were not found in most samples collected from intensive care rooms as shown in Fig. 5c, d whereas 16 S DNA, which indicates total bacteria, was found on both bed frames and walls in intensive care rooms (cycle threshold value: ~ 20–27). The cycle threshold value for the bed frame sample was 37.58, but it was considered a negative result according to the Korean criteria, which specify that positive tests are those with cycle threshold values below 35. This implies that SARS-CoV-2 did not exist in the negative pressure wards, even though they were not disinfected. Patients who rely on nasal cannula equipment or are under extubation management are more likely to limit the contamination of their sur rounding environment because they have difficulty moving and communicating.

In contrast, in single-person and double-shared rooms in which patients could move and communicate, SARS-CoV-2 was found in various environmental samples. In particular, SARS-CoV-2 was found in most samples (cell phone, toilet in lavatory, and bed frame) in double-shared rooms rather than in single-person rooms. Interestingly, the virus was found on surfaces in close proximity to (bed or toilet) or frequently used by patients (cell phone). The virus was found only on a cell phone in a single-person room in which the patient could move (cycle threshold value: 35). It is likely that this patient had used the cell phone frequently. Thus, we conclude that without disinfection, SARS-CoV-2 can often be found around patients when they move and/or speak.

## Conclusions

In the current pandemic situation, numerous studies are being conducted worldwide with molecular diagnosis technology (e.g., PCR), false-positive signals that occur because of free RNA fragments, which has already been released and cannot be distinguished from that of infectious viruses, has always presented major problems for the field of diagnostics. In this study, surfaces found in hospital wards, including surfaces of cell phones, walls, and toilets, were investigated using a novel sample preparation method that could differentiate the virus alone from free RNA fragments. Consequently, it was possible to isolate and measure only the abundance of the virus itself, not the free RNA fragments (over 99% removal of free RNA fragments were attained). We believe that this protocol and a simple kit can be directly applied to the medical and quarantine industries, including for the re-selection of asymptomatic infected patients and determination of quarantine termination for confirmed infected patients.

## Supplementary Information

**Additional file 1: Table S1.** PCR primer set of each RNA and DNA. **Figure S1.** SARS- CoV-2 RNA stability in environmental surface condition. **Figure S2.** Result of SARS-CoV-2 detection by using new sample preparation protocol and commercial protocol (identical concentration spread on the surface (1x1 m^2^)). **Figure S3.** Layout of sampling area in (a) single-person room and (b) double sharing room. **Figure S4.** Calibration curve of (a) HCoV-229E, (b) SARS-CoV-2 (triplicated).

## Data Availability

The datasets used and/or analysed during the current study are available from the corresponding author on reasonable request.
