# Supplementary material for: Discrimination and isolation of the virus from free RNA fragments for the highly sensitive measurement of SARS-CoV-2 abundance on surfaces using a graphene oxide nano surface
Source: Nano Converg. 2021 Oct 18;8:31. doi: 10.1186/s40580-021-00281-8 (PMC8521082; doi:10.1186/s40580-021-00281-8)
Supplement: Supplementary file 1 — Additional file 1: Table S1. PCR primer set of each RNA and DNA. Figure S1. SARS- CoV-2 RNA stability in environmental surface condition. Figure S2. Result of SARS-CoV-2 detection by using new sample preparation protocol and commercial protocol (identical concentration spread on the surface (1x1 m2)). Figure S3. Layout of sampling area in (a) single-person room and (b) double sharing room. Figure S4. Calibration curve of (a) HCoV-229E, (b) SARS-CoV-2 (triplicated). [file 40580_2021_281_MOESM1_ESM.docx]

**Additional Information**

**Highly sensitive measurement of SARS-CoV-2 abundance on surfaces using a graphene oxide nano surface to discriminate and isolate the virus from free RNA fragments**

Hyun Jin Yoo^†^, Yun Guang Li^†^, Wen Ying Cui^†^, Wonseok Chung^‡^, Yong-Beom Shin^‡^, Yeon-Sook Kim^§^, Changyoon Baek^†^**, and Junhong Min^†^*

^†^School of Integrative Engineering, Chung-Ang University, Heukseok-dong, Dongjak-gu, Seoul, 06974, South Korea

^‡^BioNano Health Guard Research Center, Daejeon, 34141, South Korea

^§^Division of Infectious Diseases, Department of Internal Medicine, Chungnam National University School of Medicine, Munhwa-ro 282, Jung-gu, Daejeon, 35015, South Korea

Table S1. PCR primer set of each RNA and DNA

Figure S1. SARS- CoV-2 RNA stability in environmental surface condition

Figure S2. Result of SARS-CoV-2 detection by using new sample preparation protocol and commercial protocol (identical concentration spread on the surface (1x1 m^2^))

Figure S3. Layout of sampling area in (a) single-person room and (b) double sharing room

Figure S4. Calibration curve of (a) HCoV-229E, (b) SARS-CoV-2 (triplicated)
